# Detailed registration of care in midwifery practices in the Netherlands: an opportunity for research within a healthy pregnant population

**DOI:** 10.1186/s12884-020-03053-0

**Published:** 2020-06-16

**Authors:** A. Pouwels, P. Offerhaus, A. Merkx, B. Zeegers, M. J. Nieuwenhuijze

**Affiliations:** Research Centre for Midwifery Science, Zuyd University, Maastricht, the Netherlands

**Keywords:** Pregnancy, Data collection, VeCaS

## Abstract

**Background:**

Research in maternity care is often conducted in mixed low and high-risk or solely high-risk populations. This limits generalizability to the low-risk population of pregnant women receiving care from Dutch midwives. To address this limitation, 24 midwifery practices in the Netherlands bring together routinely collected data from medical records of pregnant women and their offspring in the VeCaS database. This database offers possibilities for research of physiological pregnancy and childbirth. This study explores if the pregnant women in VeCaS are a representative sample for the national population of women who receive primary midwife-led care in the Netherlands.

**Methods:**

In VeCaS we selected a low risk population in midwife-led care who gave birth in 2015. We compared population characteristics and birth outcomes in this study cohort with a similarly defined national cohort, using Chi Square and two side t-test statistics. Additionally, we describe some birth outcomes and lifestyle factors.

**Results:**

Midwifery practices contributing to VeCaS are spread over the Netherlands, although the western region is underrepresented. For population characteristics, the VeCaS cohort is similar to the national cohort in maternal age (mean 30.4 years) and parity (nulliparous women: 47.1% versus 45.9%). Less often, women in the VeCaS cohort have a non-Dutch background (15.7% vs 24.4%), a higher SES (9.9% vs 23.7%) and live in an urbanised surrounding (4.9% vs 24.8%). Birth outcomes were similar to the national cohort, most women gave birth at term (94.9% vs 94.5% between 37 + ^0^–41+ ^6^ weeks), started labour spontaneously (74.5% vs 75.5%) and had a spontaneous vaginal birth (77.4% vs 77.6%), 16.9% had a home birth. Furthermore, 61.1% had a normal pre-pregnancy BMI, and 81.0% did not smoke in pregnancy.

**Conclusions:**

The VeCaS database contains data of a population that is mostly comparable to the national population in primary midwife-led care in the Netherlands. Therefore, the VeCaS database is suitable for research in a healthy pregnant population and is valuable to improve knowledge of the physiological course of pregnancy and birth. Representativeness of maternal characteristics may be improved by including midwifery practices from the urbanised western region in the Netherlands.

## Background

Most primary care midwives in the Netherlands are organized in independent midwifery practices in the community, and care for healthy women with uncomplicated pregnancies and births. Primary midwife-led care emphasizes the normality of the reproductive process, recognizing that pregnancy and childbirth are physiological processes. Midwives are ‘gatekeepers’ and refer women to a secondary care obstetric team if risk factors or complications arise during pregnancy, labour or after birth. Interventions such as augmentation of labour, pharmacological pain relief, continuous foetal monitoring or instrumental birth are only accessible in secondary care [1–3]. Women who start their antenatal care in primary, midwife-led care are free to choose a birth at home, in a birth centre or in the hospital, attended by their own primary care midwife. When women are referred to obstetrician-led care during the antenatal or intrapartum period because of an increased risk or complication, these women give birth in the hospital, attended by a secondary care obstetric team (hospital-based midwives, residents and obstetricians) [4].

Currently, evidence-based midwifery in Dutch maternity care is often based on national and international research performed in mixed low and high-risk or solely high-risk populations. This limits generalizability of evidence to a low-risk population of healthy, pregnant women in primary midwife-led care.

The Midwifery Science department from Zuyd University in Maastricht has set up the Midwifery Case Registration System (Verloskundig Casusregistratie Systeem, VeCaS) to address the need for research in a healthy, low-risk population. VeCaS is a database, which includes anonymized quantitative data extracted from the medical files of women receiving care in 24 Dutch midwifery practices from 2012 onwards. VeCaS contains information about each professional contact with a woman and her baby during the prenatal, natal and postnatal period. It collects data such as blood pressure, maternal weight and length, ultrasounds, abdominal palpations, birth mode, birthweight, other neonatal measures, and information about referrals and consultations with other professionals. VeCaS collects information on pregnancies and births in much more detail than available in the existing database Perined from the Netherlands Perinatal Registry. This national registry routinely collects and combines a limited number of items on antenatal, intrapartum and postnatal care in four separate national registries; one for primary midwife-led care (LVR1), one for maternity care by general practitioners (LVR1h), one for obstetrician-led care (LVR2), and one for neonatal care (LNR). Perined contains data on approximately 98% of all births in the Netherlands [5].

The midwives participating in VeCaS are instructed verbally about collection and registration of the data to improve data validity. Subsequently, together with the participating midwives, a consensus manual with definitions of the variables has been created. Midwifery practices bi-yearly receive a summary of their own registered data for feedback on their quality of registration to optimize data collection and/or registration. The VeCaS database increases with approximately 6.000 records each year. Informed consent is obtained from each woman before extracting the anonymized data from the medical file to VeCaS. On average 10 (range 0–50) women per practice per year do not provide informed consent and are not included. Ethical approval for the database was obtained from the regional Medical Research Ethics Committee Maastricht (nr 09–4-061).

Research in the VeCaS database addresses the needs for more evidence on the physiological course of pregnancy and birth. The Netherlands can provide a healthy population database since primary midwife-led care in the Netherlands is well known internationally for its low-intervention rate and its high homebirth rate. This might be of interest of other countries as well.

The aim of this study is to examine if pregnant women registered in VeCaS offer a representative reflection of the national population of women who receive primary care in midwifery practices in the Netherlands, considering maternal and pregnancy characteristics.

## Methods

### Study population

We created a study cohort that included all women registered in VeCaS who gave birth in 2015, and who received antenatal care up to at least 28 weeks of gestation in one of the 24 participating midwifery practices. Likewise, we created a national cohort using the Perined database. The study populations in both cohorts, being in primary midwife-led care at 28 weeks of pregnancy, are, by definition, considered to be low risk.

**Fig. 1:**
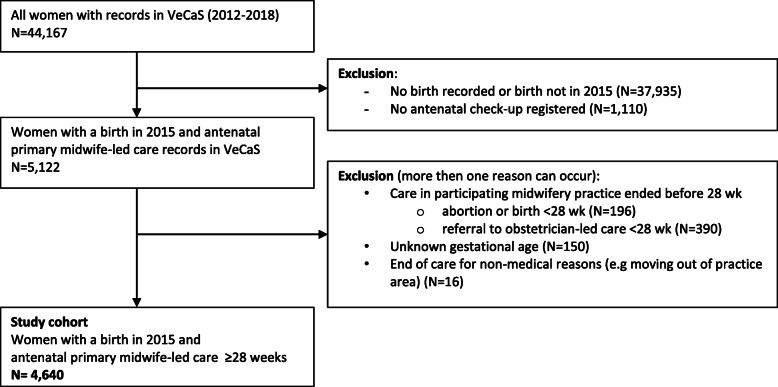
Flowchart: selection of the study population

We choose 2015, because complete data on pregnancy and birth were available in VeCaS as well as Perined at the start of the study. We excluded women who gave birth before 28 weeks or at an unknown gestational age, women who were transferred to obstetrician-led care before 28 weeks, women who had a multiple pregnancy and women whose antenatal care in the midwifery practice ended for non-medical reasons such as moving out of the practice area (see Fig. 1 for a flowchart of the VeCaS cohort).

### Analysis

We compared the VeCaS cohort with the created national cohort on age, parity, background (Dutch or non-Dutch), social economic status (SES) and level of urbanization. The two latter characteristics were based on the four digits of the postal code [6]. We used Chi Square or two side t-test statistics to test whether these differences are statistically significant. Analyses were performed with SPSS 25.

Additionally, we describe other birth and pregnancy characteristics (gestational age at birth, start of labour, mode of birth, place and level of care at birth, and birthweight) of the VeCaS cohort compared with the national cohort.

We also describe the body mass index (BMI) and smoking behaviour in the VeCaS cohort. We cannot compare these life style characteristics with the created national cohort, since these are not reliably available in the Perined database.

## Results

### Practice characteristics

**Fig. 2:**
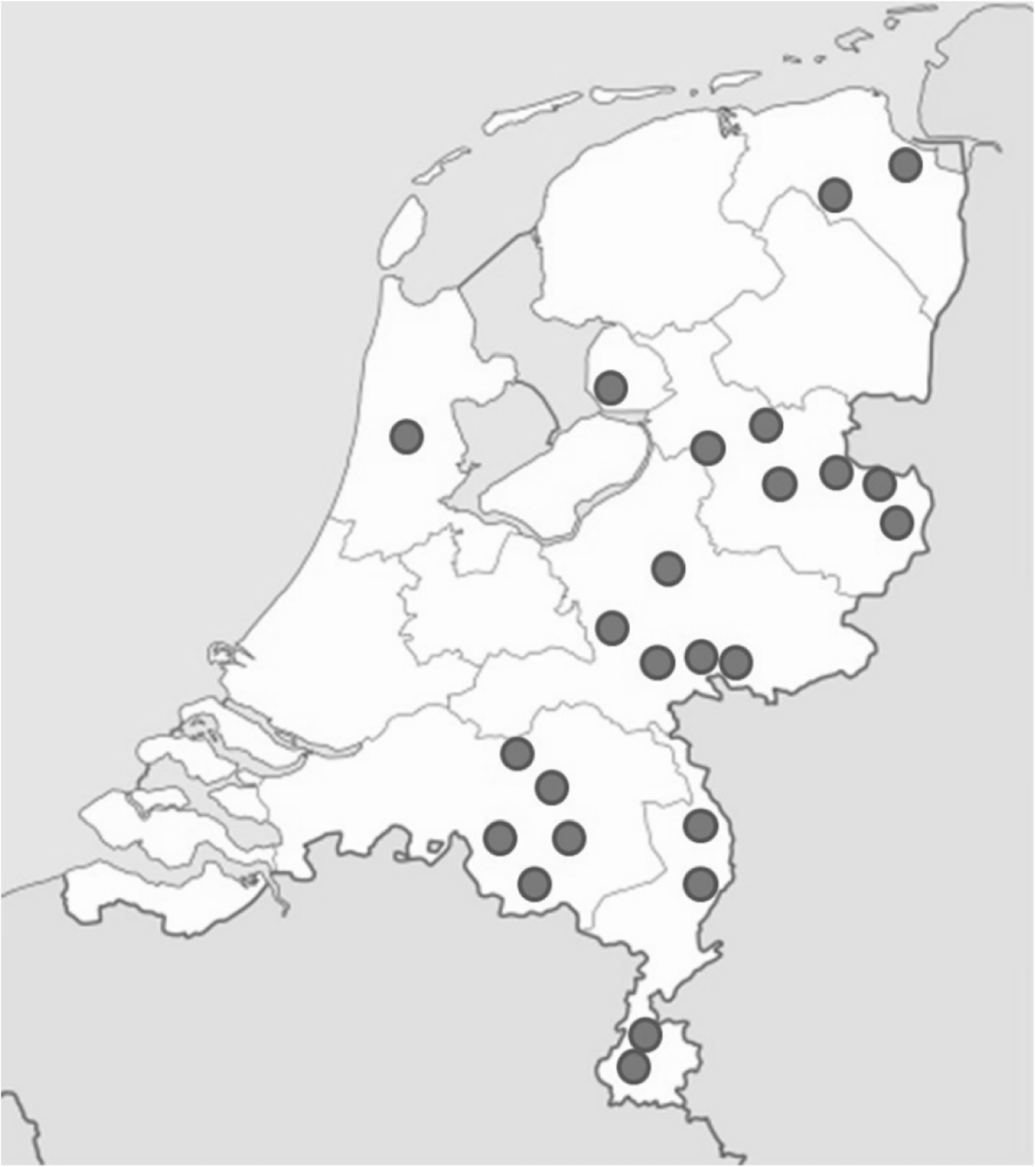
Location of 24 midwifery practices participation in VeCaS in 2015 in the Netherlands

The 24 midwifery practices participating in VeCaS (2015) were situated throughout the Netherlands (see Fig. 2). The distribution shows more practices to the southeast compared to the western part of the Netherlands. Most of the practices (*N* = 13) were located within a rural region, the remaining were located within an urban region (*N* = 5) or a combination of both (*N* = 6). The number of women cared for in the practices varied from 93 to 532, with an average of 294. Most of the practices were organized as a duo with two midwives (*N* = 4) or as a group with three midwives or more (*N* = 20). No solo practice was represented. The midwives had a mean age of 41 years (median age 38; range 22–61).

### Population characteristics

The VeCaS cohort contained records of 4640 women. Using the same inclusion criteria, the national cohort contained records of 127,818 women in 2015. Maternal age and parity showed no relevant differences between both cohorts. The mean maternal age in the VeCaS cohort and the national cohort was 30.4 years (SD 4.5, SD 4.6); 47.1% women in the VeCaS cohort were nulliparous and 52.9% multiparous, compared to 45.9% nulliparous and 54.1% multiparous women in the national cohort.

Other characteristics were distributed less equally. In VeCaS, 84.1% of the women had a Dutch background compared to 75.6% of the women in Perined. In the VeCaS cohort women with a higher SES (9.9%) were underrepresented compared to the national cohort (23.7%). Furthermore, in the VeCaS cohort most women lived in middle (32.4%) or low (23.4%) urbanized regions whereas in the Perined cohort more women lived in very high (24.8%) and high (25.6%) urbanized regions.

Lifestyle characteristics in VeCaS showed that 61.1% of the women had a normal pre-pregnancy BMI, and 81.0% did not smoke in pregnancy (see Table 1).

**Table 1:** Characteristics women in the VeCaS cohort compared to national cohort (2015)

	VeCaS cohort		National cohort		
	*N* = 4640	100%	*N* = 127,818	100%	
**Maternal age**					
All women (mean, SD)	30.4	(SD 4.5)	30.4	(SD 4.6)	*p* = 1.00
Primiparous women (mean, SD)	28.8	(SD 4.3)	28.9	(SD 4.6)	*p* = 0.3174
***Age in categories***					
All women					
< 20 yr	27	0.6%	1125	0.9%	
20–24 yr	373	8.0%	12,072	9.4%	
25–29 yr	1575	33.9%	41,138	32.2%	
30–34 yr	1802	38.8%	49,438	38.7%	
35–39 yr	748	16.1%	20,885	16.3%	
≥ 40 yr	115	2.5%	3160	2.5%	
**Parity**					
P0	2184	47.1%	58,608	45.9%	χ^2^ = 16.757, df = 2, *p* = 0.0002
P1	1723	37.1%	46,010	36.0%	
P2+	733	15.8%	23,198	18.1%	
missing	0		2		
**Background**					
Dutch	3713	84.1%	96,589	75.6%	χ^2^ = 171.058, df = 1, *p* < 0.0001
Non-Dutch	700	15.7%	31,229	24.4%	
missing	227				
**Social Economic Status (SES)**^**a**^					
Higher	455	9.9%	30,149	23.7%	χ^2^ = 499.183, df = 2, p < 0.0001
Middle	2538	55.2%	55,949	44.0%	
Lower	1606	34.9%	41,146	32.3%	
missing	41		574		
**Urbanization**^**b**^					
Very high (> 2500)	226	4.9%	31,693	24.8%	χ^2^ = 1463.241, df = 4, p < 0.0001
High (1500–2500)	894	19.4%	32,707	25.6%	
Middle (1000–1500)	1498	32.4%	23,989	18.8%	
Low (500–1000)	1080	23.4%	21,991	17.2%	
very low (100–500)	919	19.9%	17,232	13.5%	
missing	23		206		
**BMI in kg/m**^**2**^					
Low (< 18,5)	149	3.2%			
Normal (18,5-24,9)	2777	61.1%			
Overweight (25–29,9)	1088	23.9%			
Obesitas (≥30)	530	11.7%			
missing	96				
**Smoking in pregnancy**					
No	3676	81.0%			
Stopped in 1st trimester	367	8.1%			
Yes	497	10.9%			
missing	100				

^a^based on 4 digits of the postal code

^b^based on 4 digits of the postal code, number of households/km^2^

### Birth and pregnancy characteristics

Within the VeCaS cohort, most women gave birth at term (94.9% from 37^+ 0^ to 41^+ 6^ weeks). Labour mostly started spontaneously (74.5%) and most women experienced a spontaneous vaginal birth (77.4%). This is comparable with the national cohort (see Table 2). Both in the VeCaS and in the national cohort, 16.9% of the women experienced a home birth. An equal proportion of women was referred during pregnancy or labour to an obstetric team and gave birth in the hospital in secondary care: 64.7% in the VeCaS cohort and 63.9% in the national cohort. The average birthweight was 3465 g (SD 522) in the VeCaS cohort compared to 3469 g (SD 513) in the national cohort.

**Table 2:** Description of birth characteristics in the VeCaS cohort compared to national cohort (2015)

	VeCaS cohort		National cohort	
	*N* = 4640	100%	*N* = 127,818	100%
**Gestational age in weeks**				
< 37 weeks	196	4.2%	5121	4.1%
37^0^–41^6^ weeks	4403	94.9%	119,429	94.5%
≥ 42 weeks	41	0.9%	1836	1.5%
**Start of labour**				
Spontaneous	3403	74.5%	96,547	75.5%
Induction	893	19.6%	23,853	18.7%
Elective caesarean section	271	5.9%	7416	5.8%
missings	73			
**Mode of birth**				
Spontaneaous vaginal	3506	77.4%	98,846	77.6%
Instrumental vaginal	390	8.6%	10,504	8.2%
Caesarean section	634	14.0%	18,000	14.1%
missings	110			
**Place and level of care at birth**				
Home (midwife, primary care)	776	16.9%	21,632	16.9%
Birth centre (midwife, primary care)	101	2.2%	3943	3.1%
Hospital (midwife, primary care)	748	16.2%	20,528	16.2%
Hospital (obstetric team, secondary care)	2977	64.7%	81,674	63.9%
missings	38		41	
**Birthweight**				
Mean	3465	SD (522)	3469	SD (513)
**Birthweight categories**				
< 2500 g	154	3.3%	4267	3.3%
2500–4500 g	4368	94.9%	120,844	94.7%
> 4500 g	82	1.8%	2459	1.9%
missings	36		248	

## Discussion

In this study, we explored whether the pregnant women registered in VeCaS are a representative reflection of the national population of women who receive primary midwife-led care in midwifery practices in the Netherlands. Based on age and parity, the VeCaS cohort corresponds adequately with the cohort derived from the national perinatal database Perined. Both cohorts are also comparable regarding birth characteristics such as home births, spontaneous start of labour and spontaneous vaginal births. These birth characteristics reflect the healthy, low-risk profile of the VeCaS cohort.

We were not able to compare lifestyle characteristics within the VeCaS cohort, as reliable information on smoking behaviour and BMI are not available in Perined. However, national statistics suggest that non-smoking behaviour in our cohort (81.0% non-smokers) was somewhat more frequent than in the general population of women aged 25–45 years in 2015 (75.6% non-smokers), and that some more women had a normal BMI compared to women in this age category in 2015 (61.1 vs 57.9%) [7].

Women with a non-Dutch ethnic background, SES classified as higher and women who lived in a very high or high urbanized region are underrepresented within the VeCaS cohort. It is likely that the representativeness of VeCaS for the population in primary midwife-led care in the Netherlands will improve by including midwifery practices from the highly urbanized region called ‘the Randstad’ in the western region in the Netherlands. In collaboration with the Amsterdam/Groningen Academy of Midwifery (AVAG) new practices in this region are being recruited and the first records are included in the VeCaS database since 2018.

Characteristics of the participating midwifery practices show a relative young age of midwives (median age 38). This corresponds with the midwifery profession in the Netherlands, where 64% of midwives is under 40 years of age [8]. Midwifery practices organized as solo are not represented in the VeCaS database at this moment. Since only 5% of the primary care midwives in the Netherlands works in a solo practice, [8], we assume that the underrepresentation of solo midwifery practices do not affect the representativeness of the VeCaS population.

The VeCaS database is highly suitable for research within a healthy pregnant population. The information collected throughout pregnancy and childbirth within the VeCaS database creates a possibility to describe in detail characteristics of healthy pregnancies and the care that was offered. In our study cohort a population was selected who received antenatal care from their midwife up to at least 28 weeks of gestation, reflecting the population who receive primary midwife-led care in the Netherlands. The database offers the possibility to select records of women who stay in primary midwife-led care until the start of term labour - without antenatal referral to obstetrician-led care -, this group reflects a population that experienced a pregnancy without significant complications. For example, if we would select records this group of women in our study cohort (*n* = 2702; 58.2%), 86.4% of these women experienced a spontaneous vaginal birth and 28.1% a home birth (data not shown). Depending on the research question, an even healthier sub-population within the VeCaS database can also be defined, for instance only women with healthy life style characteristics. Selecting only women with a normal pre-pregnancy BMI and non-smoking behaviour provides an opportunity to investigate for example physiological changes in blood pressure, weight gain or to develop curves for birthweight appropriate for gestational age.

## Conclusion

The VeCaS database contains rich pregnancy and birth information on a population that is mostly comparable to the national population in primary midwife-led care in the Netherlands, considering maternal age, parity, gestational age, birthweight, and birth outcomes. The database represents a healthy population and therefore offers an ideal opportunity to improve knowledge on the physiological course of pregnancy and birth. By including more midwifery practices located in the highly urbanized western region of the Netherlands, representativeness of maternal characteristics such as SES, urbanization and ethnicity can be further improved in the VeCaS database.

## Data Availability

The datasets used and/or analyzed during the current study are available from the corresponding author on reasonable request.

## Abbreviations

AVAG: Amsterdam/Groningen Academy of Midwifery
BMI: Body Mass Index
SES: Social Economic Status
SPSS: Statistical Package for the Social Sciences
VeCaS: Verloskundig Casusregistratie Systeem (Midwifery Case Registration System)
